# Could NSP5a3a be a target for head and neck cancer?

**DOI:** 10.18632/oncotarget.172

**Published:** 2010-09-13

**Authors:** Martyn K. White, Kamel Khalili

**Affiliations:** Department of Neuroscience, Center for Neurovirology, Temple University School of Medicine, Philadelphia, PA, USA

The novel structural proteins (NSPs) are encoded by a family of related but distinct cDNAs expressed from a single gene [1]. This gene corresponds to locus HCMOGT-1 on chromosome 17p11.2 and encodes NSP5a3a and three other splice variants. NSP5a3a is highly expressed in some tumor cell lines and in the testis but not in other normal body tissues [1]. Further studies revealed that NSP5a3a interacts with the nucleolar phosphoprotein B23, which plays multiple roles in cell growth and proliferation [2] and with hnRNP-L [3].

In this issue of *Oncotarget*, D'Agostino and Giordano investigate the effect of over-expressing NSP5a3a in head and neck squamous cell carcinoma (HNSCC) cell lines [4]. HNSCC is one of the 10 most common cancers worldwide and the overall survival rate for HNSCC is still low even with the latest innovations in both basic and clinical research [5]. Thus, there is an urgent requirement to identify novel targets for HNSCC. The present study indicates that NSP5a3a may represent such a potential target. When NSP5a3a was over-expressed in HN30 HNSCC cells, there was a robust induction of apoptosis. The authors went on to investigate the molecular mechanisms involved in the effects of NSP5a3a in HN30 and FADU HNSCC cells and found no changes in p53, B23, Bax or caspase 3 but a possible involvement of p73.

These data raise some interesting questions: “could NSP5a3a be a target for the therapy of HNSCC?” and “could agonists of NSP5a3a be developed and used in combination with chemotherapy and/or radiotherapy for HNSCC?”. The development of such therapies likely requires a more detailed elucidation of the mechanism of action of NSP5a3a involving characterizing NSP5a3a biochemically and the development of inhibitors/agonists for the signal transduction pathways involved. In conclusion, these data are an interesting starting point to investigate the role of this protein in cancer in general and HNSCC in particular.
